# Supplementary material for: Medaka (Oryzias latipes) initiate courtship and spawning late at night: Insights from field observations
Source: PLoS One. 2025 Feb 12;20(2):e0318358. doi: 10.1371/journal.pone.0318358 (PMC11819472; doi:10.1371/journal.pone.0318358)
Supplement: S1 Table — (PDF) [file pone.0318358.s001.pdf]

Supplementary information

S1 Table. Temperatures, sunrise time, and sunset time in Gifu, Japan, from July 25 to August 10, 2023.

Date	Average temperature (°C)	Maximum temperature (°C)	Minimum temperature (°C)	Sunrise time	Sunset time
July 25th	29.0	34.1	25.1	4:56	19:03
July 26th	31.5	39.1	27.4	4:56	19:02
July 27th	30.6	39.4	26.3	4:57	19:01
July 28th	30.7	37.3	25.1	4:58	19:01
July 29th	30.6	36.5	27.4	4:59	19:00
July 30th	30.9	37.5	26.3	4:59	18:59
July 31st	31.2	36.9	27.9	5:00	18:58
August 1st	31.5	37.2	27.8	5:01	18:57
August 2nd	30.1	36.2	25.2	5:02	18:56
August 3rd	29.8	34.7	27.5	5:02	19:56
August 4th	30.9	36.7	27.2	5:03	18:55
August 5th	30.4	36.4	26.4	5:04	18:54
August 6th	30.2	36.2	26.6	5:05	18:53
August 7th	29.3	33.8	24.8	5:05	18:52
August 8th	30.7	36.1	27.7	5:06	18:51
August 9th	29.4	34.4	26.6	5:07	18:50
August 10th	30.4	35.7	26.2	5:08	18:49
