# Supplementary material for: Medaka (Oryzias latipes) initiate courtship and spawning late at night: Insights from field observations
Source: PLoS One. 2025 Feb 12;20(2):e0318358. doi: 10.1371/journal.pone.0318358 (PMC11819472; doi:10.1371/journal.pone.0318358)
Supplement: S2 Table — (PDF) [file pone.0318358.s002.pdf]

Supplementary information

S2 Table. Number of medaka (*Oryzias latipes*) recorded during the breeding season in Gifu, Japan.

Time	Total number of medaka observed	Mean ± SE of the number of medaka observed per video	Minimum medaka observed in one video	Maximum medaka observed in one video	Number of video samples analyzed
21:00–22:00	269	8.97±1.29	1	27	30
22:00–23:00	247	8.23±1.20	1	31	30
23:00–0:00	254	8.47±1.28	1	25	30
0:00–1:00	361	12.03±1.31	2	35	30
1:00–2:00	377	12.57±1.04	3	28	30
2:00–3:00	358	11.93±1.57	2	43	30
3:00–4:00	340	11.33±1.23	1	27	30
4:00–5:00	270	9.31±1.23	1	27	29
